# ‘We Are the Cornerstone of This Hospital’: First‐Hand Accounts of Expert‐By‐Experience Practices in Forensic Psychiatry in Finland

**DOI:** 10.1111/inm.70079

**Published:** 2025-06-09

**Authors:** Katja Lumén, Olavi Louheranta, Lauri Kuosmanen

**Affiliations:** ^1^ Department of Forensic Psychiatry University of Eastern Finland, Niuvanniemi Hospital Kuopio Finland; ^2^ Department of Nursing Science University of Eastern Finland Kuopio Finland

**Keywords:** expert‐by‐experience, forensic psychiatry, patient participation, thematic analysis, user involvement

## Abstract

Experts‐by‐experience can help care personnel in planning, producing and evaluating care and promoting the patient perspective in care. Patients in forensic psychiatry rarely influence core processes in hospitals, but involving them as experts‐by‐experience in various assignments has become a desirable way of making forensic psychiatric services more patient‐centred. This is the first study to outline the forms that expert‐by‐experience work takes in Finnish forensic hospitals. It summarises experiences of such work from the perspectives of experts‐by‐experience themselves and the staff who work with them. We interviewed 19 experts‐by‐experience and 18 professionals who work with them to reveal the current situation of expert‐by‐experience activities in Finnish forensic psychiatric hospitals. We used inductive thematic analysis to explore their experiences. Our findings identify five main themes: a transformative effect, the resources as a contribution, motivation and achievement, interaction and co‐operation and identification as validation. The results from this study show that a wide range of expert‐by‐experience tasks are assigned to current and former patients in these hospitals, but experts‐by‐experience have not yet gained a formal position in most of them. We identified several benefits of expert‐by‐experience work for different stakeholders, along with challenges to the implementation of EBE practices. We hope that this study will promote the development of expert‐by‐experience work in forensic psychiatric hospitals. A COREQ Checklist was applied.

## 1 Introduction

Patient participation and expert‐by‐experience practices in health care services have been recommended (Daya et al. 2020), but genuine co‐production and involving patients in all phases of their care are rare in psychiatric institutions (Pilgrim 2018). One way of promoting patient participation and the patient perspective in care is to provide services in collaboration with experts‐by‐experience (EBEs). In this study, we examined how expert‐by‐experience practices have thus far been organised, received and experienced in Finnish forensic psychiatric hospitals. We hope that this paper will offer valuable insights into current expert‐by‐experience practice and promote realistic new possibilities in this *field*.

## 2 Background

In recent decades, EBEs have been employed in psychiatry, including forensic psychiatry (Livingston et al. 2013), in an attempt to serve the principle of user involvement in service production (Neech et al. 2018) and incorporate a recovery orientation into the care system (Gordon and Bradstreet 2015). Implementing EBE work has been challenged by inadequate resourcing (Gordon and Bradstreet 2015; Livingston et al. 2013) and issues concerning trust, the mental stability of the EBE peer and their attitudes towards treatment (Walde et al. 2021). Patient participation and experiences of being heard are important (Møllerhøj and Os Stølan 2018) and can increase patient satisfaction with forensic psychiatric care (Schröder et al. 2016) but they require the active input and assistance of staff, particularly in forensic psychiatric hospitals (Livingston et al. 2013; Selvin et al. 2021). Shared decision making in forensic psychiatric hospitals is complicated by the need to balance risk, care, safety and privacy (Pilgrim and Rogers 2008; Zhao et al. 2024). Staff and patients do not always take the same views about which forms of care are helpful (Marklund et al. 2020). The level and form of patient participation in psychiatry has been rationalised through the evaluated mental state or capacity of the participating patient (Tambuyzer et al. 2014), but no standards have been set as to who can become an EBE and when.

Patient‐centred care and patient participation are normally discussed in terms of the individual patient's perspective (Håkansson Eklund et al. 2019; Jones and Pietilä 2020; Jørgensen et al. 2023), but EBE work aims to include patients as partners in organisational actions at various levels. In this sense, the EBE is not just an expert in their own distinctive experience but is seen as a representative of patients and clients more generally (Jones and Pietilä 2020; Lindström 2024). Whereas Håkansson Eklund et al. (2019) describe the mutual factors in concepts ‘patient‐centred’ and ‘person‐centred’ care, they emphasise that person‐centred care proposes the patient is rather a rational actor than merely a person suffering from a psychiatric condition. Vidaurrazaga Aras et al. (2024) link the idea of person‐centred care closely to patient participation in forensic psychiatry. Noorani (2013) suggests that the service user movement (EBE work) started as a radical survivor movement but has been reduced to one which uses EBEs as underlings of official power structures. The organisation holds the power to decide who can act as an EBE and under which conditions (Brosnan 2012) and due to this power, the genuine collaborative action can become impossible (Tambuyzer et al. 2014). The ladder of participation (Arnstein 1969) distinguishes eight levels of participation ranging from nonparticipation, through degrees of tokenism (being heard but not being taken into account) to, at its highest level, actual citizen power. The challenge of genuine participation in forensic, psychiatric, highly secure institutions is complex (Livingston et al. 2013). Treatment is typically open‐ended, involuntary and takes place in an environment which gives patients limited opportunities to leave or make decisions about their own issues (Söderberg et al. 2022). Participation as a genuine form of power (Arnstein 1969) is therefore problematic. This can place a significant mental burden on the patient (Møllerhøj and Os Stølan 2018). Involving EBE peers in psychosocial interventions has been shown to improve patients' commitment to care (e.g., Castelein et al. 2008). Incorporating EBEs into delivering care does not necessarily promote patients' own aspirations, but it has improved their willingness to adhere to institutional requirements. Further, the use of EBE peers has promoted personal recovery (Livingston et al. 2013) and supported patient empowerment (Leamy et al. 2011). In psychiatry in general, the inclusion of EBEs has become the desired way of service production through both legal stipulations and user‐led actions (Chinman et al. 2014; Jones and Pietilä 2020; Noorani 2013). Still, a rather recent study in Germany showed that EBEs as peers are not very common in German forensic psychiatry (Walde et al. 2021).

EBEs have been successfully involved in the planning (Vojtila et al. 2021), delivery (Vojtila et al. 2021; Walde et al. 2021) and evaluation of psychiatric care, and in associated education (Happell et al. 2022) and research (Fusar‐Poli et al. 2022; Livingston et al. 2013; Tambuyzer et al. 2014), as well as published literature alongside professionals (Drennan and Alred 2013; Fusar‐Poli et al. 2022) providing a patient perspective based on their lived experience. EBEs themselves have also benefitted from these assignments (Happell et al. 2022). In this study, we aimed to delineate the past and current EBE practices in Finnish forensic and to provide positive guidance to the future development of these practices by providing insight into current experiences. Our research questions were:

What work has been undertaken in Finnish forensic psychiatric hospitals with the involvement of EBEs?

What do EBEs and the staff who work with them consider to have been the most important aspects, influences and impacts of this work to date?

## 3 Methods

### 3.1 Design

#### 3.1.1 Participants

We used purposive sampling to reach informants who had knowledge of previous and present EBE work in Finnish forensic hospitals and invited all experts and staff working with them to participate in the interview study. We reached participants in all five Finnish organisations which provide forensic care in designated units through local contacts. According to our estimation, approximately 50 EBEs were assigned to different tasks at the time of the interviews. 19 EBEs (17 men and two women) and 18 staff members (17 women and one man) who work with them agreed to participate. 17 of the EBEs reported that they were or had been treated in forensic psychiatric hospitals, 15 of them as forensic patients. Two EBEs were willing to participate but could not be included due to their schedules and plans; three provided preliminary consent but declined before the interview. One could not participate due to language issues. All professionals attended the interviews as planned.

The ages of the EBEs' ranged from 27 to 62 years (mean 39.5) and they had been treated in forensic psychiatry from two to more than 10 years. Most (*n* = 15) reported that their psychiatric illness has started before the age of 30. Five had obtained a degree after elementary school. The professionals' ages ranged between 29 and 63 years. They had psychiatric work experience from three to 39 years (mean 22.5). Most were nurses (registered nurse, licensed practical nurse, ward manager, assistant ward manager, nursing manager, mental health nurse). The professions occupational therapist, chief physician, psychologist, rehabilitation instructor and project worker were also reported. The professionals' educational background varied from vocational to PhD studies. The interviews with the EBEs lasted on average 26 min (ranging from 11 to 56 min) and with the professionals 62 min (ranging from 27 min to 2 h and 41 min).

#### 3.1.2 Ethical Considerations

The Regional Medical Research Ethics Committee of the Wellbeing Services County of North Savo provided us with an affirmative statement concerning the study design, and each organisation authorised our study according to its own protocol.

All participants were given both written and verbal explanations of the purpose and conduct of the study weeks before taking part, and again at the start of each interview. All participants provided written consent. They were informed of the possibility to decline the invitation to participate or interrupt the interview at any time without any consequences. The interviewees received no remuneration.

#### 3.1.3 Data Collection

Face to face (with 18 experts and six professionals) and on‐line (with one expert and 12 professionals) interviews took place between December 2021 and August 2023, and all conversations were recorded with the participants' consent. Our interviewees were all involved in EBE activities, which were defined and confirmed at the beginning of each interview. The main researcher (K.L.) interviewed all but one of the participants and was not familiar with them from before. One EBE was interviewed by a local worker (T.L.) and this worker had met the interviewee before the study took place.

The professionals and EBEs were asked about their participation in different forms of EBE activities, how does a person become an EBE, whether the EBEs receive some education, remuneration or support, what works in EBE activities and its' challenges, who profits from EBE work, are the EBEs' thoughts and ideas heard, what are the requirements for EBEs and which new activities could be undertaken?

#### 3.1.4 Analysis

We took a social constructionist stance, adopting Goffman's (1961) conception of forensic psychiatric hospitals as setting limits within which all human activities take place. Although Goffman is often classified as a symbolic interactionist, (see e.g., Gonos 1977) his idea of the totalitarian institution reflects a structuralist posture. According to Goffman, individuals in society submit themselves to, and make choices, interact and take actions following, the rules of the superior power structure, although patients in closed institutions (such as forensic psychiatric hospitals) do not choose to submit to superior power structures (e.g., those of the institution) (Pilgrim and Rogers 2008) but must still adhere to them. Patients' roles are given and predetermined, not self‐made (Lock 2010; Pilgrim and Rogers 2008) although activities involving EBEs demonstrate the possibility for an individual's role to shift from that of a patient to that of an EBE (Pilgrim and Rogers 2008).

**FIGURE 1:**
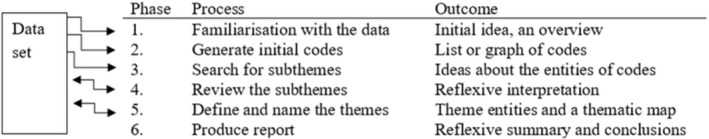
Analysis schedule.

We followed Braun and Clarke (2006) in conducting the thematic analysis in phases as shown in Figure 1. We were interested in EBE work on a very practical level and thus chose to analyse the text for its manifest meanings. Our analysis was necessarily inductive (Braun and Clarke 2006) as there was no previous research available concerning EBE work in Finnish forensic psychiatry.

In Phase 1 the interviews were transcribed verbatim by the first author (K.L.), who then collected the data set (descriptions of EBE work) from the data corpus (all interview transcripts). To immerse ourselves in the data before moving to the next phase (Braun and Clarke 2006) we then re‐read and made notes on the data, forming an overview and initial ideas about its contents. In Phase 2 we coded each data item (interview) and compiled them into a single text file of data extracts relevant to the research questions (Braun and Clarke 2006).

During Phase 3 we searched for correspondence between the meaning of the codes and the defined initial subthemes. These were reviewed in Phase 4 of the analysis and compared with the data set and data items to ensure that our analysis was logical. In Phase 5 we summarised the subthemes into meaningful themes, revisiting the codes for trustworthiness, before proceeding to producing the report in Phase 6 (Braun and Clarke 2006, 2021). Phases 4 and 5 were a joint effort between the first and third authors (K.L.& O.L.).

**FIGURE 2:**
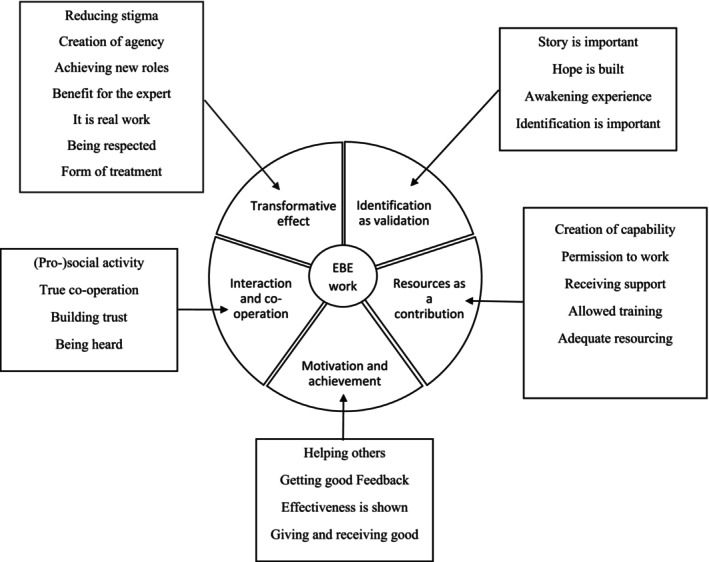
Subthemes and themes.

A clear description of the data analysis process adds to the trustworthiness of thematic analysis and qualitative research in general. The logic and phases of the analysis process and the interpretations made during it must be presented to readers (Nowell et al. 2017). We illustrate our analysis schedule in Figure 1 and our reasoning for grouping subthemes and themes in Figure 2. A COREQ Checklist (COnsolidated criteria for REporting Qualitative research) was utilised to improve the rigour and trustworthiness of our study (Tong et al. 2007).

## 4 Results

We delineated the various assignments performed by the EBE workers. These assignments take place during several phases of service production, including planning, delivery and evaluation, and are summarised as follows:

*Peer support* takes place in groups or individually. Group meetings with staff are frequently held and may touch on addiction issues or comprise psychoeducational interventions. Some hospitals allow patients and EBEs to meet in private without the presence of staff members. EBE peers can accompany their fellow patients to different activities and support them in acquiring new skills and are also available for confidential conversation. The major benefits of such peer‐to‐peer work include support to both the EBE client and the EBE peer worker in their respective recovery processes.

EBEs *disseminate information* to various audiences. These may encompass students, patients' family members, hospital visitors, nurses and the hospital board. EBEs may find it difficult to prepare and deliver lectures or speeches due to having problems with verbal communication or a lack of IT skills, and they often require support from staff. The reward for EBEs is being heard and their audience obtaining new insights into the patient perspective.

*Patient councils* are organised at ward and hospital levels. As with peer support, these gatherings are sometimes arranged by EBEs alone, but often members of staff are present. The councils aim to bring up the patient perspective on care and promote improvements to treatment, conditions and the quality of care in the hospital. Through such councils, EBEs and staff have successfully minimised frictions in how patients and staff interact, and suggestions arising from them have led to unique improvements in the hospital such as visitor facilities (establishing a separate restroom and apartment for overnight use with patients), changes to the ward environment, enhanced patient privacy and new opportunities to spend time in the hospital yard.

*Work groups and executive teams* have invited EBE patients to share their expertise relating to new facilities both before and after they have been built. Numerous staff work groups intended to diminish the use of coercion, or to improve work therapy or nutrition in the hospital, have also involved EBEs as permanent members. For EBEs, understanding organisation‐level issues can be challenging. Nonetheless, the EBEs expressed that they experienced being genuinely heard and included.

Our thematic analysis resulted in five themes which are important to understanding EBE work as a whole and its' meaning in Finnish forensic psychiatric hospitals. EBE work can be comprehended through its *transformative effect, resources as a contribution, motivation and achievement, interaction and co‐operation and identification as validation*.

### 4.1 Transformative Effect

Through EBE work the patient can acquire a new role as an expert. EBE work is perceived as real work with goals, both requiring and building capacity, and can also be seen as a form of treatment due to its positive effects on different actors. It can promote the EBE's personal recovery and self‐esteem, confidence, open‐mindedness and trustworthiness in the eyes of the staff, and help them learn to regulate their workload and burden.

I believe it has been relevant to their recovery journey. This is about participation, ability and raising their area of expertise… It has been very beneficial for their skills, being a part of this, for sure some of them get braver and dare to work in other places. (Professional 17)

The influences and impacts of EBE work include reducing stigma, creating agency, supporting new roles, increasing respect for the EBE's expertise, and benefiting the expert as well as the people receiving support or information. EBE work was seen to be effective and worthwhile for various stakeholders:

If I was a nurse, I would use these EBEs quite often in the patient meetings because sometimes the nurse doesn't even notice that they are stuck… With an EBE present new things can open up. (Professional 18)

Through peer work in particular, EBEs process their life stories and gain new insights into their past, present and future, including the hardship caused by severe mental illness and criminal offending. The following excerpt expresses these effects:

This has been one of the best things during my recovery journey supporting me right from the start and up to the present moment, in the sense that this EBE work has supported my own well‐being. (EBE 19)

I can help friends or those I meet, and I gain self‐confidence. I guess it's best when I can help someone. (EBE 4)

### 4.2 Resources as a Contribution

EBE work could be seen as a tokenistic form of patient participation and modern psychiatric care, not truly supported and appreciated throughout the organisation. EBE work receives positive and encouraging feedback from many directions, but the resources for it are often insufficient. Staff associated this issue with the still inconsistent and unofficial status of EBE activities. Staff working with EBEs shared the trouble they had finding enough time to take care of all the tasks associated with organising EBE activities:

This lack of time, these challenges are upsetting, many of us are exhausted at times, just can't handle it… (Professional 12)

Many EBEs need repeated help with writing their invoices or reminders to come to meetings. These issues are seen as normal for people with a severe mental illness. In some cases, EBEs were given tasks that were too burdensome and had to withdraw themselves from performing them. The level of capability and recovery (internal resources of the EBE) needed to perform various EBE assignments was evaluated differently. EBEs were supported through counselling, peer support and education. With these forms of support the EBEs could utilise their personal histories as the root of their expertise. An example of this was:

…[EBEs' capacity depends on]…a stable mental state, as mentioned, and their own experience. They have climbed up from the bottom and of course want to and are able to share. They have the resources to receive what comes up from others in the group, and of course they have counselling where they can vent. (Professional 1)

Forensic psychiatric EBEs can also encounter prejudice and stigma, both outside and inside their institution. In some hospitals, it was only possible to become a peer after completing an education programme within the institution itself, although any such education was seen as valuable. Educational programmes outside of hospitals may be too demanding for forensic psychiatric patients to complete, if they involve months of training and a final assignment.

Getting them into that education… they couldn't… For sure, there were some obstacles, fear, stigma. …This group of patients was not well known at higher levels in the organisation. (Professional 16)

### 4.3 Motivation and Achievement

Motivation stems from good feedback, sense of achievement and gaining recognition. The main motivation for EBEs was helping others by supporting their peers and promoting improvements in hospital care. Peer workers specifically stated that they hoped to help others, but the EBE's client is not the only one to benefit from the meetings:

Well, these discussions between the peer and the client are confidential, but they give building blocks to the client when they can pour their heart out to the peer. (EBE 8)

I can also see these things in myself, and I get that satisfaction for me as well, it's kind of repetition. (EBE 3)

Receiving good feedback was a clear source of motivation. When patients' and EBEs' initiatives were successful it motivated both the EBEs and the professionals.

It has been many years… now we got one good thing done when we got that guest room here, it was really cool. (EBE 3)

Some EBEs gained recognition from staff members as official collaborators and were granted salaries as well as a badge or an outfit. Attitudes towards remuneration varied between hospitals:

After the experience I gained about so many things, that's why I started, and I intend to keep working as a peer outside the hospital. And the salary was a partial cause. (EBE 9)

### 4.4 Interaction and Co‐Operation

Key goals for the EBE work included being heard and giving patients a voice. Ward councils were launched to improve co‐operation between staff and patients and to support EBE patients' pro‐social abilities.

The work [of the ward council] has brought the patients and staff together. There was a lot of confrontation, but due to the council we now do things together. (Professional 9)

During council meetings, staff needed to abandon their position as experts and actively listen to EBE patients' experience. Open discussion in council meetings allowed trust to develop, and this facilitated better interaction. During peer discussions, trust meant not divulging the content of meetings to staff unless particular issues were raised. This could put the EBE peer in a difficult intermediary position:

We talk about joys and sorrows and have rules about suicidal or drug issues, we need to tell the staff about those. But we can talk about drugs in a constructive way. (EBE 3)

Pro‐social activities could be learned in co‐operation with others and social activities were seen an important part of recovery.

Basically, things which are important to them but not personal, that has been our goal, to change antisocial behaviour into more prosocial, but of course they themselves also benefit. (Professional 12)

Peers spoke about how they encouraged the patients they met to speak to the staff more freely. Although peer support was seen to require a rather stable mental state, peer group activities were also possible for EBEs who could not communicate clearly but could organise activities for others as well as themselves.

### 4.5 Identification as Validation

EBEs' life stories are the main source of their expertise and, as such, extremely important and valid:

Through strong comprehension and understanding my own history, I can pretty well see what the cause of everything has been. (EBE 13)

For an outsider, coming to a forensic hospital can be distressing. An EBE peer with a similar history can create hope and serve as an example of overcoming difficulties. A peer can address difficult subjects in a different, more honest way than staff can. The shared experiences are true and validated.

They need to have tangible experience, deeper, that they have been in deep waters. (Professional 7)

Especially in the beginning it can be difficult to accept treatment and motivate yourself into rehabilitation so maybe it is more credible that there is someone who has their own experience of the illness. (Professional 5)

Having an EBE who did not have a forensic history was seen as problematic, because they lacked deep understanding of having a severe mental illness and being hospitalised in a forensic institution.

One of the EBEs told us:

Maybe [it's] that I have been here for a long time and in a way know what it's like on a closed ward, I know about seclusion and restraints and these things. (EBE 12)

## 5 Discussion

We were able to reach all forensic psychiatric organisations in Finland and describe the EBE activities undertaken in these institutions. We found large differences in what and how has been done within EBE work. We also discovered the advantages as well as challenges in current and past EBE activities. Experiences of EBE work can be perceived through five themes with different meanings, motivations and perceptions for different stakeholders.

The findings of this study reflect the core components of personal recovery for forensic psychiatric patients: personal development, social inclusion, redemption and overcoming (Livingston et al. 2013; Lumén et al. 2024). EBEs said that all of these aspects of recovery are present in their own experiences of EBE work and in the support they provide to their fellow patients. The EBE work helps the EBEs themselves to recover and build capacity (Happell et al. 2022; Neech et al. 2018). However, support and consideration are needed to ensure that EBEs are involved in activities and education that are suited to their capacity and that the workload does not become unbearable (Jones and Pietilä 2020; Livingston et al. 2013; Neech et al. 2018). This is particularly important when EBEs are still hospitalised themselves and have a dual role as both a patient and an expert. Building hope and promoting social networks through peer work has proven beneficial for patients who suffer from long‐term and difficult cognitive impairments (Castelein et al. 2008). Sharing mutual experiences, being an example of recovery, and just being a friend were valued benefits for both EBE peers and their clients in our study. Peer support can promote recovery and reduce internalised stigma (Livingston et al. 2013) while being granted the opportunity to share life and recovery stories with others can promote EBEs' empowerment (Jones and Pietilä 2020; Lindström 2024).

Limited recognition of the importance of EBEs and inadequate resourcing (Gordon and Bradstreet 2015) were often mentioned as problems, and interviewees linked these issues to the sometimes tokenistic status of EBE activities. Involving patients as EBEs in hospitals calls for a new stance from staff (Gordon and Bradstreet 2015); EBEs need the active support of staff to genuinely participate and fulfil their assignments (Livingston et al. 2013; Selvin et al. 2021). In this study, staff emphasised a conscious decision to pursue equality in joint councils with EBEs, and EBEs said that they felt appreciated and valued. Their views were heard and there were no topics which could not be touched upon while co‐operating with staff. The EBEs were not seeking to become parallel to the professionals—each has their own part to play in promoting the well‐being of patients—but EBE activities were referred to as a form of treatment. Some patients are reluctant to share their thoughts openly with staff (Zhao et al. 2024) but are willing to discuss them with EBE peers. When patients do not trust staff members, they can lose hope and resign from treatment (Marklund et al. 2020).

From the perspective of Arnstein's (1969) model of citizen participation, the involvement of EBEs in Finnish forensic psychiatry includes elements which range from manipulation to perceived partnership. Interviewees felt that using EBEs as ambassadors of staff opinion was both unethical and misses opportunities to learn from EBEs and promote an authentic patient perspective. However, EBEs in all of the hospitals in this study were required to approve of the care on offer (Walde et al. 2021). Different tasks enable patients to engage in organisational assignments in different ways but both EBEs and professionals stated that, in the best examples, they perceive true delegated power (Arnstein 1969) and partnership arising in EBE work, especially in patient councils. From the perspective of Goffman's conceptions of a totalitarian institution (1961), the patient councils in particular shift the traditional power associated with expertise from staff to patients and stretch the boundaries of the hospital as they provide new opportunities for EBE patients to speak out, be heard, and become socially active in new ways. However, due to the context, the professionals are always responsible for decisions and their consequences (Arnstein 1969). Being heard and giving patients a voice were central goals of the EBE work studied, and these can promote patient participation even if not all their wishes are granted (Goffman 1961; Marklund et al. 2020; Söderberg et al. 2022).

The literature on EBEs and patient participation concentrates primarily on the individual level. We found a wide range of EBE tasks in our study, ranging from personal peer meetings to participation in executive teams. It was stated that some of the tasks are unofficial, and this possibly explains the difficulties of making EBE work a distinguishable part of forensic psychiatric treatment. There is still some resistance to giving patients in forensic hospitals verifiable status as experts.

## 6 Conclusion

Based on our findings, we suggest that EBE work should be made an official part of forensic psychiatric treatment, granted a stronger status in hospitals, and promoted through education, counselling, rewards, and adequate resources and support for all participants. EBE activities should be arranged by each organisation to ensure that they fit both patients' and organisational needs. Joint research by EBEs and professionals into forensic psychiatric care is still lacking but could be undertaken in future as a new form of EBE work.

## 7 Relevance for Clinical Practice

This paper is the first to offer a summary of EBE practices in Finnish forensic psychiatry. We found differences between EBE assignments in different hospitals and hope to stimulate further learning from current experience. At its best, EBE work promotes recovery and the quality of care, co‐operation, and overall well‐being, offering hope and positive experiences to all who are involved in it.

## Conflicts of Interest

The authors declare no conflicts of interest.

## Data Availability

The authors have nothing to report.

## References

[inm70079-bib-0001] Arnstein, S. R. 1969. “A Ladder of Citizen Participation.” Journal of the American Institute of Planners 35, no. 4: 216–224. 10.1080/01944366908977225.

[inm70079-bib-0002] Braun, V. , and V. Clarke . 2006. “Using Thematic Analysis in Psychology.” Qualitative Research in Psychology 3, no. 2: 77–101. 10.1191/1478088706qp063oa.

[inm70079-bib-0003] Braun, V. , and V. Clarke . 2021. Thematic Analysis. SAGE Publications, Ltd.

[inm70079-bib-0004] Brosnan, L. 2012. “Power and Participation: An Examination of the Dynamics of Mental Health Service‐User Involvement in Ireland.” Studies in Social Justice 6, no. 1: 45–66. 10.26522/ssj.v6i1.1068.

[inm70079-bib-0005] Castelein, S. , R. Bruggeman , J. T. Van Busschbach , et al. 2008. “The Effectiveness of Peer Support Groups in Psychosis: A Randomized Controlled Trial.” Acta Psychiatrica Scandinavica 118, no. 1: 64–72. 10.1111/j.1600-0447.2008.01216.x. 18595176

[inm70079-bib-0006] Chinman, M. , P. George , R. H. Dougherty , et al. 2014. “Peer Support Services for Individuals With Serious Mental Illnesses: Assessing the Evidence.” Psychiatric Services (Washington, D.C.) 65, no. 4: 429–441. 10.1176/appi.ps.201300244. 24549400

[inm70079-bib-0007] Daya, I. , B. Hamilton , and C. Roper . 2020. “Authentic Engagement: A Conceptual Model for Welcoming Diverse and Challenging Consumer and Survivor Views in Mental Health Research, Policy, and Practice.” International Journal of Mental Health Nursing 29, no. 2: 299–311. 10.1111/inm.12653. 31538723 PMC7328715

[inm70079-bib-0008] Drennan, G. , and D. Alred . 2013. Secure Recovery. Willan. 10.4324/9780203129173.

[inm70079-bib-0009] Fusar‐Poli, P. , A. Estradé , G. Stanghellini , et al. 2022. “The Lived Experience of Psychosis: A Bottom‐Up Review Co‐Written by Experts by Experience and Academics.” World Psychiatry 21, no. 2: 168–188. 10.1002/wps.20959. 35524616 PMC9077608

[inm70079-bib-0010] Goffman, E. 1961. Asylums: Essays on the Social Situation of Mental Patients and Other Inmates. Anchor Books.

[inm70079-bib-0011] Gonos, G. 1977. “‘Situation’ Versus ‘Frame’: The ‘Interactionist’ and the ‘Structuralist’ Analyses of Everyday Life.” American Sociological Review 42, no. 6: 854–867. 10.2307/2094572.

[inm70079-bib-0012] Gordon, J. , and S. Bradstreet . 2015. “So if We Like the Idea of Peer Workers, Why Aren't We Seeing More?” World Journal of Psychiatry 5, no. 2: 160–166. 10.5498/wjp.v5.i2.160. 26110117 PMC4473487

[inm70079-bib-0013] Håkansson Eklund, J. , I. K. Holmström , T. Kumlin , et al. 2019. “‘Same Same or Different?’ A Review of Reviews of Person‐Centered and Patient‐Centered Care.” Patient Education and Counseling 102, no. 1: 3–11. 10.1016/j.pec.2018.08.029. 30201221

[inm70079-bib-0014] Happell, B. , A. O'Donovan , J. Sharrock , T. Warner , and S. Gordon . 2022. “Understanding the Impact of Expert by Experience Roles in Mental Health Education.” Nurse Education Today 111: 105324. 10.1016/j.nedt.2022.105324. 35278940

[inm70079-bib-0015] Jones, M. , and I. Pietilä . 2020. “Personal Perspectives on Patient and Public Involvement – Stories About Becoming and Being an Expert by Experience.” Sociology of Health & Illness 42, no. 4: 809–824. 10.1111/1467-9566.13064. 32072657

[inm70079-bib-0016] Jørgensen, K. , M. Hansen , T. G. Andersen , M. Hansen , and B. Karlsson . 2023. “Healthcare Professionals' Experiences With Patient Participation in a Mental Healthcare Centre: A Qualitative Study.” International Journal of Environmental Research and Public Health 20, no. 3: 1965. 10.3390/ijerph20031965. 36767331 PMC9916001

[inm70079-bib-0017] Leamy, M. , V. Bird , C. Le Boutillier , J. Williams , and M. Slade . 2011. “Conceptual Framework for Personal Recovery in Mental Health: Systematic Review and Narrative Synthesis.” British Journal of Psychiatry 199, no. 6: 445–452. 10.1192/bjp.bp.110.083733. 22130746

[inm70079-bib-0018] Lindström, J. 2024. “Survival Stories as Access to Society: Narrative Research on Experts by Experience With a History of Crime and Substance Abuse.”

[inm70079-bib-0019] Livingston, J. D. , A. Nijdam‐Jones , S. Lapsley , C. Calderwood , and J. Brink . 2013. “Supporting Recovery by Improving Patient Engagement in a Forensic Mental Health Hospital: Results From a Demonstration Project.” Journal of the American Psychiatric Nurses Association 19: 132–145. 10.1177/1078390313489730. 23690284

[inm70079-bib-0020] Lock, A. 2010. Social Constructionism: Sources and Stirrings in Theory and Practice. Cambridge University Press.

[inm70079-bib-0021] Lumén, K. , O. Louheranta , and L. Kuosmanen . 2024. “Forensic Psychiatric Patients' Experiences of Personal Recovery: A Wilsonian Concept Analysis.” Journal of Forensic Nursing 20, no. 2: 103–112. 10.1097/JFN.0000000000000477. 38315513 PMC11882176

[inm70079-bib-0022] Marklund, L. , T. Wahlroos , G. E. Looi , and S. Gabrielsson . 2020. “‘I Know What I Need to Recover’: Patients’ Experiences and Perceptions of Forensic Psychiatric Inpatient Care.” International Journal of Mental Health Nursing 29, no. 2: 235–243. 10.1111/inm.12667. 31642598

[inm70079-bib-0023] Møllerhøj, J. , and L. Os Stølan . 2018. “‘First and Foremost a Human Being…’: User Perspectives on Mental Health Services From 50 Mentally Disordered Offenders.” Nordic Journal of Psychiatry 72, no. 8: 593–598. 10.1080/08039488.2018.1502352. 30261792

[inm70079-bib-0024] Neech, S. G. B. , H. Scott , H. M. Priest , E. J. Bradley , and A. E. Tweed . 2018. “Experiences of User Involvement in Mental Health Settings: User Motivations and Benefits.” Journal of Psychiatric and Mental Health Nursing 25, no. 5–6: 327–337. 10.1111/jpm.12466. 29753313

[inm70079-bib-0025] Noorani, T. 2013. “Service User Involvement, Authority and the ‘Expert‐By‐Experience’ in Mental Health.” Journal of Political Power 6, no. 1: 49–68. 10.1080/2158379X.2013.774979.

[inm70079-bib-0026] Nowell, L. S. , J. M. Norris , D. E. White , and N. J. Moules . 2017. “Thematic Analysis.” International Journal of Qualitative Methods 16, no. 1: 3384. 10.1177/1609406917733847.

[inm70079-bib-0027] Pilgrim, D. 2018. “Co‐Production and Involuntary Psychiatric Settings.” Mental Health Review Journal 23, no. 4: 269–279. 10.1108/MHRJ-05-2018-0012.

[inm70079-bib-0028] Pilgrim, D. , and A. Rogers . 2008. “Asylums: The Social Situation of Mental Patients and Other Inmates.” Journal of Health Services Research & Policy 13, no. 1: 47–49. 10.1258/jhsrp.2007.006066.18806192

[inm70079-bib-0029] Schröder, A. , K. Lorentzen , E. Riiskjaer , and L.‐O. Lundqvist . 2016. “Patients' Views of the Quality of Danish Forensic Psychiatric Inpatient Care.” Journal of Forensic Psychiatry & Psychology 27, no. 4: 551–568. 10.1080/14789949.2016.1148188.

[inm70079-bib-0030] Selvin, M. , K. Almqvist , L. Kjellin , and A. Schröder . 2021. “Patient Participation in Forensic Psychiatric Care: Mental Health Professionals' Perspective.” International Journal of Mental Health Nursing 30, no. 2: 461–468. 10.1111/inm.12806. 33098186 PMC7984362

[inm70079-bib-0031] Söderberg, A. , M. Wallinius , C. Munthe , M. Rask , and U. Hörberg . 2022. “Patients' Experiences of Participation in High‐Security, Forensic Psychiatric Care.” Issues in Mental Health Nursing 43, no. 7: 683–692. 10.1080/01612840.2022.2033894. 35130107

[inm70079-bib-0032] Tambuyzer, E. , G. Pieters , and C. Van Audenhove . 2014. “Patient Involvement in Mental Health Care: One Size Does Not Fit All.” Health Expectations: An International Journal of Public Participation in Health Care and Health Policy 17, no. 1: 138–150. 10.1111/j.1369-7625.2011.00743.x. 22070468 PMC5060706

[inm70079-bib-0033] Tong, A. , P. Sainsbury , and J. Craig . 2007. “Consolidated Criteria for Reporting Qualitative Research (COREQ): A 32‐Item Checklist for Interviews and Focus Groups.” International Journal for Quality in Health Care 19, no. 6: 349–357. 10.1093/intqhc/mzm042. 17872937

[inm70079-bib-0034] Vidaurrazaga Aras, V. , E. Alexiou , T. Nilsson , A. Wolf , and S. Olausson . 2024. “Factors Influencing Patient Participation in Inpatient Forensic Psychiatric Care—A Mixed‐Method Systematic Review.” International Journal of Forensic Mental Health 23: 440–455. 10.1080/14999013.2024.2388091.

[inm70079-bib-0035] Vojtila, L. , I. Ashfaq , A. Ampofo , D. Dawson , and P. Selby . 2021. “Engaging a Person With Lived Experience of Mental Illness in a Collaborative Care Model Feasibility Study.” Research Involvement and Engagement 7, no. 1: 5. 10.1186/s40900-020-00247-w. 33419484 PMC7796603

[inm70079-bib-0036] Walde, P. , C. Benz , and B. Völlm . 2021. “Implementation of a Peer Support Worker in a Forensic Hospital in Germany.” European Psychiatry 64, no. S1: S24. 10.1192/j.eurpsy.2021.88. PMC10030960 36970261

[inm70079-bib-0037] Zhao, J. , H. Bolshaw‐Walker , and N. Z. Hilton . 2024. “Engaging Forensic Psychiatry Patients in Health‐Care Decision Making.” Lancet Psychiatry 11, no. 3: 165–167. 10.1016/S2215-0366(23)00427-3. 38237617

